# Exploratory Phase II Multicenter, Open-Label, Clinical Trial of ST266, a Novel Secretome for Treatment of Persistent Corneal Epithelial Defects

**DOI:** 10.1167/tvst.11.1.8

**Published:** 2022-01-07

**Authors:** Bennie H. Jeng, Pedram Hamrah, Ziv Z. Kirshner, Benjamin C. Mendez, Howard C. Wessel, Larry R. Brown, David L. Steed

**Affiliations:** 1 Department of Ophthalmology and Visual Sciences, University of Maryland School of Medicine, Baltimore, Maryland, USA; 2 Department of Ophthalmology, Tufts University School of Medicine, Boston, Massachusetts, USA; 3 Noveome Biotherapeutics, Inc., Pittsburgh, Pennsylvania, USA

**Keywords:** persistent epithelial defect, PED, cornea, ST266

## Abstract

**Objective:**

An exploratory phase II, multicenter, open-label, clinical trial (NCT03687632) was conducted to evaluate the safety and effectiveness in treating persistent corneal epithelial defects (PEDs) with ST266, a proprietary novel multi-cytokine platform biologic solution secreted by cultured Amnion-derived Multipotent Progenitor (AMP) cells.

**Methods:**

Subjects with a PED were treated with ST266 eye drops 4 times daily for 28 days, then followed for 1 week. Safety was assessed by monitoring of adverse events (AEs) and serious adverse events (SAEs). Efficacy was assessed by measuring the area of the PED by slit lamp biomicroscopy. Tolerability of ST266, percentage of eyes with complete healing, reduction in area of the epithelial defect, and maintenance of a reduction in the area of the epithelial defect 7 days after treatment were recorded.

**Results:**

Thirteen patients were enrolled into the trial at one of eight sites. The first patient withdrew after 5 days. The remaining 12 patients with PEDs with median duration of 39 days (range = 12 to 393 days) completed treatment. Ten of the 12 eyes had been refractory to treatment with various conventional therapies prior to enrollment. After 28 days of treatment, there was a significant decrease in mean PED area compared with baseline (66.4% ± 35.3%, *P* = 0.001). At follow-up, 1 week after completion of treatment, on day 35, the PED area was further reduced by 78.8% ± 37.5% (*P* = 0.01) compared with baseline. During 28 days of treatment, 5 eyes (41.7%) had complete wound closure. There were no AEs of concern thought to be related to the drug, and no SAEs were noted.

**Conclusions:**

In this trial, we found ST266 eye drops might promote corneal epithelization, thereby reducing the PED area, including in refractory cases in a wide range of etiologies. ST266 was well-tolerated by most patients.

## Introduction

The highly innervated cornea responds to environmental changes to maintain the cornea's unique structure and transparency.^1^ Although epithelial injuries of the cornea generally heal quickly,^2^ some do not heal due to poor innervation, and this results in persistent defects. Corneal epithelial defects may be considered persistent (persistent corneal epithelial defects [PEDs]) when they have not healed in 10 to 14 days.^3^^,^^4^ Failure of the cornea to re-epithelialize makes it susceptible to infection, stromal ulceration, scarring, and even perforation, all of which can cause loss of vision.

As there are various etiologies of PEDs, therapy should address the underlying causes, such as neurotrophic keratopathy, infectious keratitis, limbal stem cell deficiency, or postsurgery complications. Treatment of a PED often begins with lubrication using preservative-free artificial tears, bandage soft contact lenses, pressure patching, debridement of the edges of the defect, scleral lenses, and/or tarsorrhaphy.^4^^–^^7^ Autologous serum eye drops and other blood-derived products^8^^–^^12^ have been used with some success, but varying concentrations of serum (from 20–100%) have been used with there being no standard dilution and rarely an attempt to identify and quantify any cytokines, including growth factors in the serum that promotes epithelial healing.

The novel secretome called ST266 (formerly known as amnion-derived cellular cytokine solution [ACCS]) was developed and produced under good manufacturing practice (GMP) conditions by Noveome Biotherapeutics, Inc. using a proprietary population of cells known as amnion-derived multipotent progenitor (AMP) cells. The AMP cells are cultured using a serum free minimal media in bioreactors and the conditioned culture media is collected over several weeks. The culture media is then further processed through filtration steps to remove certain cellular components and other debris before storage of the bulk drug product. ST266 is then quality control tested to ensure lot to lot reproducibility using a series of tests that include a panel of quantitative ELISA's, purity and cell-based potency assays. Only lots that fall within the strict specifications of these rigorous tests are used for preclinical and clinical studies. The final drug product is not concentrated and is devoid of extracellular vesicles. ST266 contains analytes at nanogram and picogram concentrations similar to levels that naturally occur in the body.^13^ Efforts to isolate individual analytes that correlate to efficacy in preclinical animal models are underway. However, in contrast to the conventional one analyte/one activity model for drug efficacy, evidence suggests that multiple analytes working synergistically likely account for the positive biological effects demonstrated in multiple preclinical studies.^14^ The presence of a subset of cytokines and growth factors present in ST266 and known in the literature to effect corneal cells can be found in Table 1.^15^^–^^27^

**Table 1.** Cytokines Identified in ST266 Drug Product With Known Corneal Cell-Related Activity

Cytokine	MW, Kd	Corneal Cell Related Activity
**PDGF-BB**	25.4	PDGF-BB induces rapid wound closure, proliferation, high motility, and late myofibroblast differentiation.^15^
**VEGF**	19.2	VEGF contributes to the cellular wound closure in multiple corneal cell layers.^16^
**EGF**	6.4	EGF shows more rapid healing of traumatic epithelial defects in a human trial.^17^
**Angiogenin**	14	Angiogenin reduces immune inflammation in human corneal fibroblast cells.^18^
**TIMP1**	21	TIMP-1 and TIMP-2 mediate corneal regenerative effects.^19^
**TIMP2**	85.8	TIMPs enhance the spreading of the corneal epithelium and proliferation of corneal epithelial cells.^20^
**DKK-3**	36.3	Dkk-3 inhibits Wnt/β-catenin signaling inhibition of Wnt/β-catenin signaling promotes epithelial differentiation of mesenchymal stem cells.^21^^,^^22^
**GDF-15**	24.6	GDF-15 is not fully clear, but it seems to have a role in regulating inflammatory pathways and to be involved in regulating apoptosis, cell repair and cell growth.^23^ GDF15 coordinates tolerance to inflammatory damage through regulation of triglyceride metabolism.^24^
**IGFBP-2**	36	IGFBP-2 and IGFBP-3 function in corneal fibroblast differentiation and myofibroblast proliferation.^25^
**IGFBP-3**	31	IGFBP-3 regulates growth control and intracellular receptor localization in the corneal epithelium.^25^
**PAI-1**	45	PAI-1 facilitates both epithelial adhesion and migration.^26^
**Osteonectin**	35	SPARC accelerated corneal epithelial wound healing and promoted the proliferation.^27^

In preclinical models, ST266 applied topically has been shown to have a positive impact on epithelial healing of the skin and the cornea. ST266 improved dermal epithelial healing in meshed human partial thickness xenografts in athymic “nude” rats.^28^ Further, in a partial thickness scald burn model in guinea pigs, ST266-treated animals had a significant increase in epithelialization compared to controls. Histology showed excellent regeneration of the epidermis with rete ridge formation.^29^ In addition, in a model of dermal healing in swine given streptozotocin to induce diabetes, healing was accelerated with a significantly thicker epidermis and more cell layers and rete ridges in the ST266 treated wounds, compared with saline controls.^30^ Moreover, in a rabbit model of corneal healing, ST266 treatment facilitated corneal re-epithelialization and was shown to be anti-inflammatory.^31^ Additionally, ST266 has been shown to be neuroprotective,^32^^–^^34^ improves impaired wound healing,^35^^,^^36^ and is anti-inflammatory.^32^^,^^35^^–^^37^

To date, phase I and II clinical trials have demonstrated ST266 to be safe and well tolerated in individuals with dry eye disease^38^ and allergic conjunctivitis.^39^ Because it is composed of multiple cytokines and growth factors, ST266 represents a potential novel biologic for treatment of PEDs. To determine whether there may be improved epithelial healing in the human cornea, similar to what was observed in rodent skin, diabetic porcine skin, and rabbit corneas, an exploratory phase II open label clinical study of ST266 for the treatment of PED was conducted.

## Materials and Methods

In an exploratory phase II multicenter, open-label clinical trial, the safety and effectiveness of ST266 in healing PEDs was tested (NCT03687632). Institutional review board (IRB) approval was obtained at each investigational site, and all participating institutions (listed in the acknowledgments section) adhered to the tenets of the Declaration of Helsinki. Patients were recruited from June 2019 until July 2020. All subjects provided written informed consent prior to enrollment. Each patient received ST266 eye drops in the study eye 4 times daily and was followed weekly for 28 days, then returned for a 7 day post-treatment follow-up visit.

Subjects aged 18 years or older with a PED of any size, measurable by slit-lamp and photography, were enrolled. Subjects who were actively being treated with cenegermin (Oxervate), autologous serum drops, amniotic membranes, bandage contact lens, and/or systemic steroids at greater than the equivalent of 10 mg of prednisone daily were excluded. Previous treatment with amniotic membranes or bandage contact lens was acceptable, if removed 24 hours before enrollment into the study, whereas systemic corticosteroids or immunosuppressants required a 7-day washout period to qualify for enrollment. Patients with uncontrolled eyelid or acute ocular infection, bullous keratopathy, corneal melting, recurrent corneal erosion, limbal blood vessel ischemia greater than 75% of the circumference of the limbus, or defects resulting from an alkali burn of the cornea, as determined by the investigator, were also excluded. Outside of exclusionary medications, patients were not restricted in concomitant treatment. Common ocular medications observed included topical antibiotics, steroids, and lubricating drops.

At baseline, patients underwent standard ophthalmic assessments, including best corrected visual acuity, tonometry, slit-lamp and dilated fundus examinations, corneal fluorescein staining, and serial measurements of the size of the corneal epithelial defects: two maximum linear dimensions perpendicular to each other were used to calculate the area of the PED by approximation formula of (a * b * π/4) [mm^2^].^40^ In addition, slit-lamp photography images were used to calculate PED area by a masked central reader (author B.H.J.) using ImageJ software (version 1.53a). Patients were instructed to distill one drop of ST266 to their eye four times a day and were provided the necessary supplies. Study visits occurred once a week for 5 weeks with an additional mid-week visit in the first 2 weeks.

The percentage of patients with complete healing of the PED at any time during the 28 days of treatment with ST266 and percent change in area from the baseline to last treatment visit on day 28 were followed. Maintenance of healing 7 days after the end of treatment, and safety and tolerability of ST266 were also recorded.

All statistical analyses were conducted using GraphPad Prism (version 8.0.2). For effect of treatment, a one-sample *t*-test was used to analyze differences in the area of the PED between baseline (day 1), end of treatment (day 28), and 1 week post-treatment follow-up (day 35), with statistical significance defined at *P* < 0.05. A scatter plot was used to identify correlation between calculated PED area to ImageJ derived area, and a Bland-Altman plot was used to analyze the agreement between the two measurement methods. For safety, a two-sided, paired *t*-test was conducted on intraocular pressure comparing baseline with days 28 and 35.

## Results

Thirteen subjects were enrolled, and 12 subjects (7 women and 5 men) completed the study. One subject withdrew on day 5 as the patient did not want to continue with an experimental drug and was not included in the data analysis. The mean age of the subjects was 70.7 ± 10.5 years. The ages of the PEDs ranged from 12 to 393 days with a median of 39 days. Demographic information, medical history, ocular condition, and additional current medications are summarized in Table 2.

**Table 2.** Patient Demographics, Previous Therapies, and Concomitant Medication

Demographics							
Patient	Age	Sex	Race/Ethnicity	Underlying Diagnosis	PED Age, Days	Previous Therapies^a^	Concomitant Medications^b^
1	76	F	Caucasian/non-Hispanic	Cicatricial pemphigoid, diabetes	48	Amniotic membrane topical steroids	Topical antibiotics antiviral topical steroids immunosuppressants lubricating drops
2	75	F	Caucasian/non-Hispanic	Neurotrophic keratopathy, unknown etiology	21	Amniotic membrane bandage contact lens topical steroids	Topical antibiotics topical steroids immunosuppressants
3	68	F	Caucasian/non-Hispanic	Herpetic keratitis, diabetes	393	Topical steroids	Topical antibiotics antiviral topical steroids immunosuppressants lubricating drops
4	56	F	Caucasian/non-Hispanic	Neurotrophic keratopathy, unknown etiology	36	Cenegermin autologous serum amniotic membrane bandage contact lens punctal plugs topical steroids	Topical antibiotics antiviral topical steroids lubricating drops
5	73	M	Caucasian/non-Hispanic	Neurotrophic keratopathy, diabetic	12	Autologous serum amniotic membrane bandage contact lens punctal plugs topical steroids	Topical antibiotics topical steroids lubricating drops
6	68	M	Caucasian/Hispanic	Status post-retinal surgery and diabetic	70	Bandage contact lens topical steroids	Topical antibiotics topical steroids lubricating drops
7	72	F	Caucasian/non-Hispanic	Recent infectious corneal ulcer	29	Amniotic membrane topical steroids	Topical antibiotics antiviral
8	50	M	African American/non-Hispanic	Status post-retinal surgery and diabetic	22	None	Topical antibiotics lubricating drops
9	86	M	Caucasian/non-Hispanic	Recent infectious corneal ulcer	44	None	Topical antibiotics
10	82	F	Caucasian/non-Hispanic	Herpes zoster	56	Autologous serum amniotic membrane bandage contact lens topical steroids	Topical antibiotics antiviral lubricating drops
11	63	M	Caucasian/non-Hispanic	Herpes zoster	12	Bandage contact lens	Topical antibiotics
12	79	F	Caucasian/non-Hispanic	Neurotrophic keratopathy, unknown etiology	41	Autologous serum topical steroids	Topical antibiotics topical steroids lubricating drops

a Previous therapies evaluated in patient medical history included amniotic membrane, bandage contact lens, autologous serum, punctal plugs, cenegermin, and topical steroids.

b Any length of concomitant medication was included. Concomitant medications evaluated included topical antibiotics and steroids, antivirals, immunosuppressants, and lubricating eye drops.

Eleven of 96 visits did not have photographs available for image analysis due to poor resolution or due to social distancing and stay-at-home guidelines during the coronavirus disease 2019 (COVID-19) pandemic. However, linear measurements were collected for all patients (*n* = 12) between visit 1 (day 1) and visit 8 (day 28), with only one measurement missing from visit 9 (day 35).

**Figure 1.**
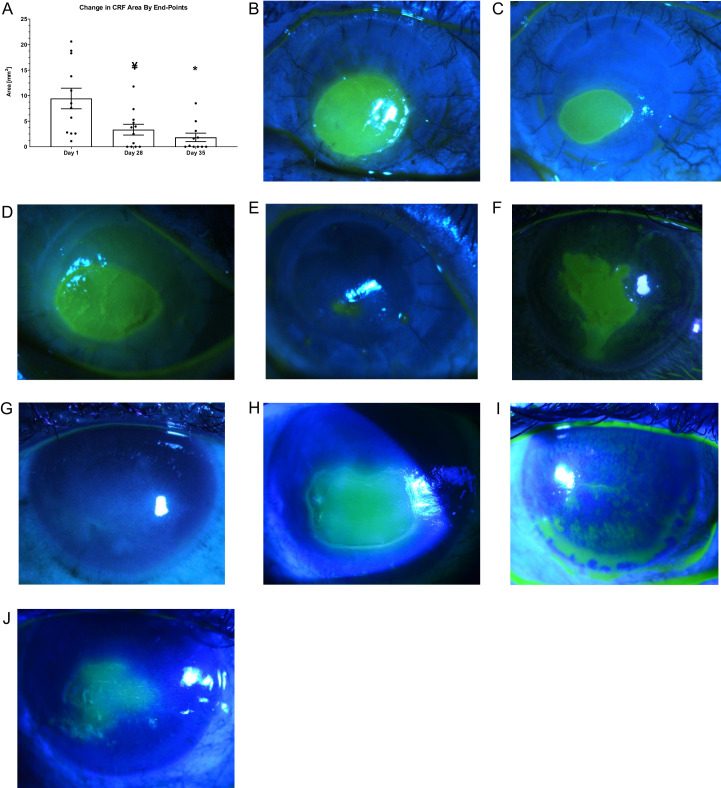
(**A**) Effect of ST266 eye drop treatment on mean area of PED at baseline (day 1), end of treatment (day 28, end of week 4) and at 1-week post-treatment follow-up (day 35, end of week 5). The mean area of PED was significantly smaller after 28 days of ST266 treatment and at day 35 compared to baseline **A**. ¥ *P* < 0.001 and * *P* < 0.01 by one sample *t*-test compared to day 1 baseline. Data shown as mean ± SEM. PED wound area visualized in patients by fluorescein staining (**B, D, F**) at baseline (day 1) and (**C, E, G**) at 1 week follow-up post-ST266 treatment (day 35) for subjects 2, 8, and 9, respectively. PED wound for subject 6 (**H**) at baseline, and (**I**) at day 28 demonstrating complete healing, but then breakdown at (**J**) day 35. Fluorescein staining images were acquired across visits and used to calculate wound area using ImageJ (version 1.53a) software.

All 12 evaluated patients showed a response with measurable re-epithelization, and, in some cases, closure of the PED by the end of 28 days of treatment with ST266 eye drops. The mean area of the PEDs at baseline was 9.45 mm^2^ ± 7.0 mm^2^ (range = 1.1 mm^2^ to 20.6 mm^2^). The mean area of the PEDs on day 28 was 3.4 mm^2^ ± 3.7 mm^2^ (range = 0.0 mm^2^ to 11.8 mm^2^). There was a 66.4% ± 35.3% reduction in area of the PEDs by day 28. The effect of ST266 treatment on PED size was statistically significant at day 28, compared with baseline (t(11) = −5.8, *P* = 0.001; Fig. 1). During 28 days of treatment, 5 eyes (41.7%) had complete wound closure. It was also noted that by day 28 all 12 PEDs had a smaller PED area by at least 3.9% and up to 100% as compared to their individual baselines.

At day 35, one week after the end of treatment, the mean PED area was 1.5 mm^2^ ± 2.7 mm^2^ (range = 0.0 mm^2^ to 8.5 mm^2^) and was significantly reduced compared with PED area at baseline (t(10) = −9.18, *P* = 0.01; see Fig. 1). This represents a 78.8% ± 37.5% reduction in PED area compared to baseline (see Fig. 1). Additionally, the mean PED area was significantly smaller at day 35 compared with day 28 (t(10) = −1.8146, *P* = 0.0498). This represented a 24.3% ± 47.7% reduction in PED area. At day 35, there were 10 of 12 eyes that had smaller PED areas, whereas one eye had a larger PED area compared with the individual baseline (patient 3). One additional subject was unevaluable at day 35 due to COVID-19 restrictions (patient 10).

**Figure 2.**
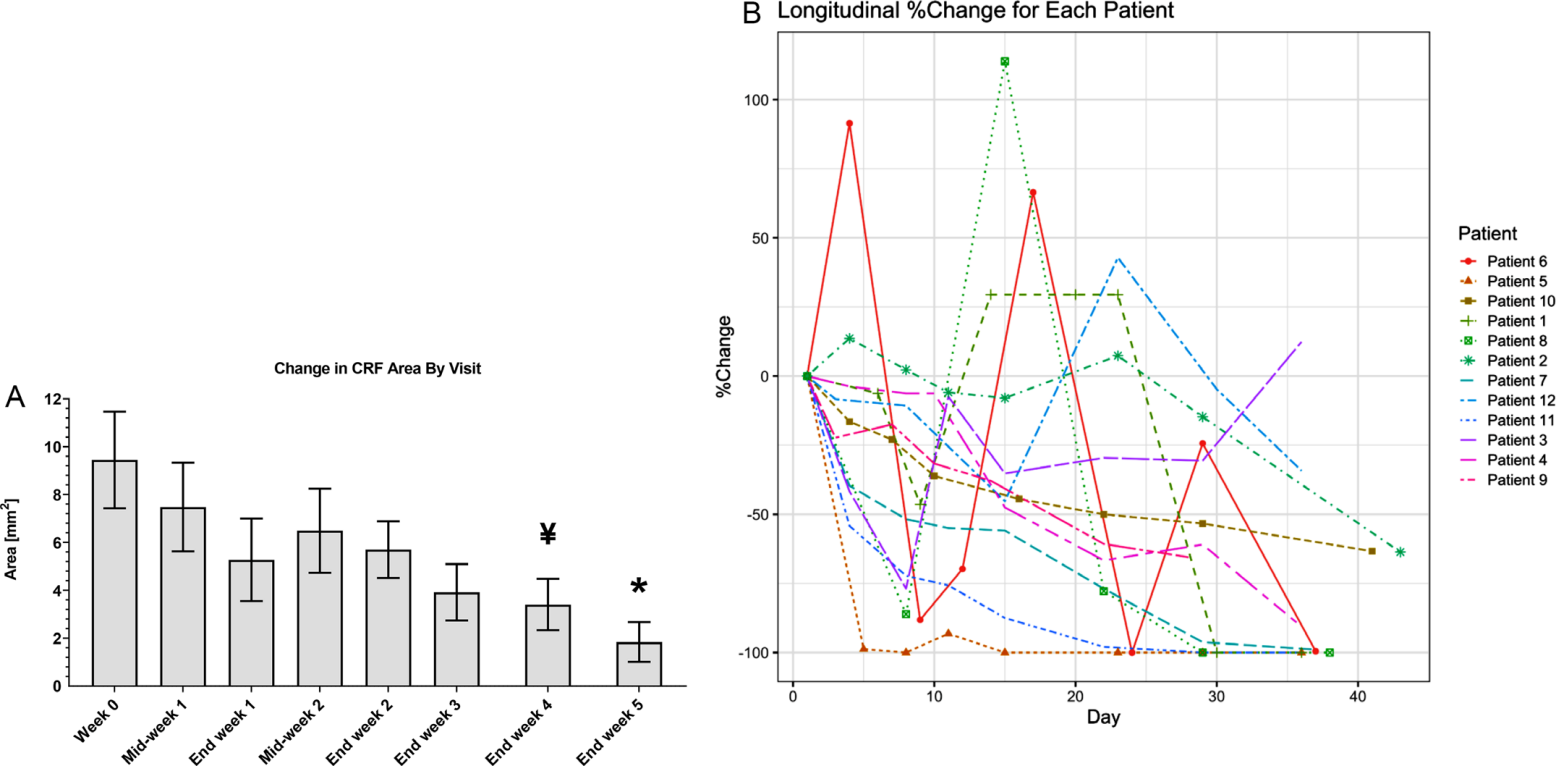
Change in area of PEDs during topical ST266 treatment and follow-up. (**A**) Change in mean area of PED over time during and after ST266 eye drop treatment. (**B**) Mean area of PED across patient visits. Longitudinal plot of change in PED area by time per each patient in this study. ¥ *P* < 0.001 and * *P* < 0.01 by one sample *t*-test compared to day 1 (baseline). Data shown as mean ± SEM.

Representation of the mean PED area across time showed a gradual decrease by the end of treatment compared to baseline (Fig. 2A). A longitudinal analysis illustrated that 11 out of 12 patients showed a continuous reduction in PED area across time (Fig. 2B) and some of these patients showed a transient episode of noticeable increase in PED area (*n* = 6). Further analysis revealed that five out of six patients with noticeable transient episodes of increase in the PED area were diabetic, whereas six out of seven non-diabetic patients had continuous reduction in PED area (Fig. 3). No significant pattern was identified in an exploratory data analysis by categorical grouping of patients (e.g. by demographic data and medical history as described in Table 1). However, a possible correlation was observed by grouping diabetic and non-diabetic patients. Linear regression identified significant fit of the duration of ST266 treatment on the re-epithelization in non-diabetic patients (see Fig. 3B).

**Figure 3.**
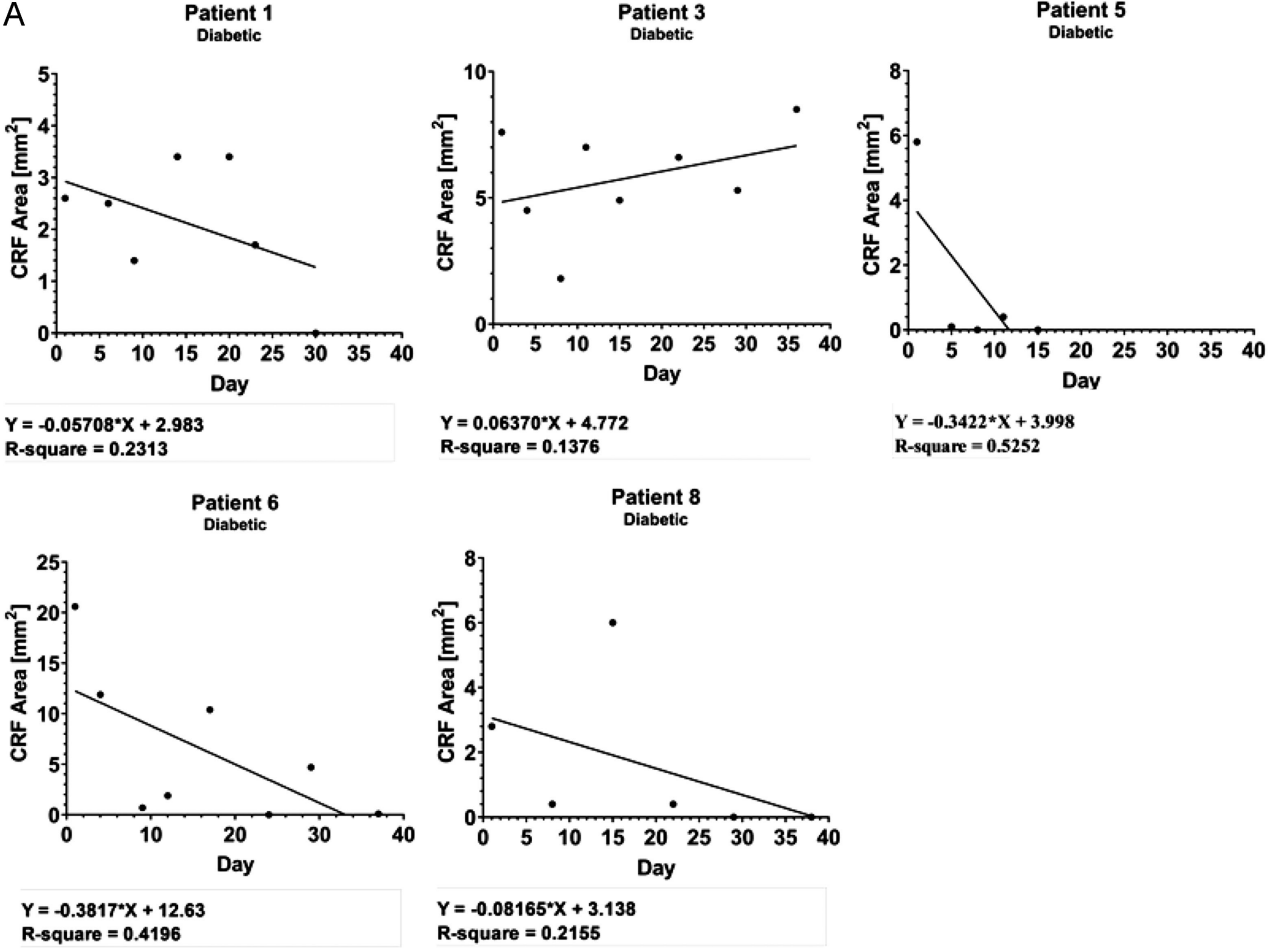
Reduction in the size of PED of individual patients during treatment with ST266 and at follow-up. Kinetics of corneal wound healing by ST266 treatment is illustrated in (**A**) diabetic and (**B**) non-diabetic patients by fitment of a linear regression line.

**Figure 3.**
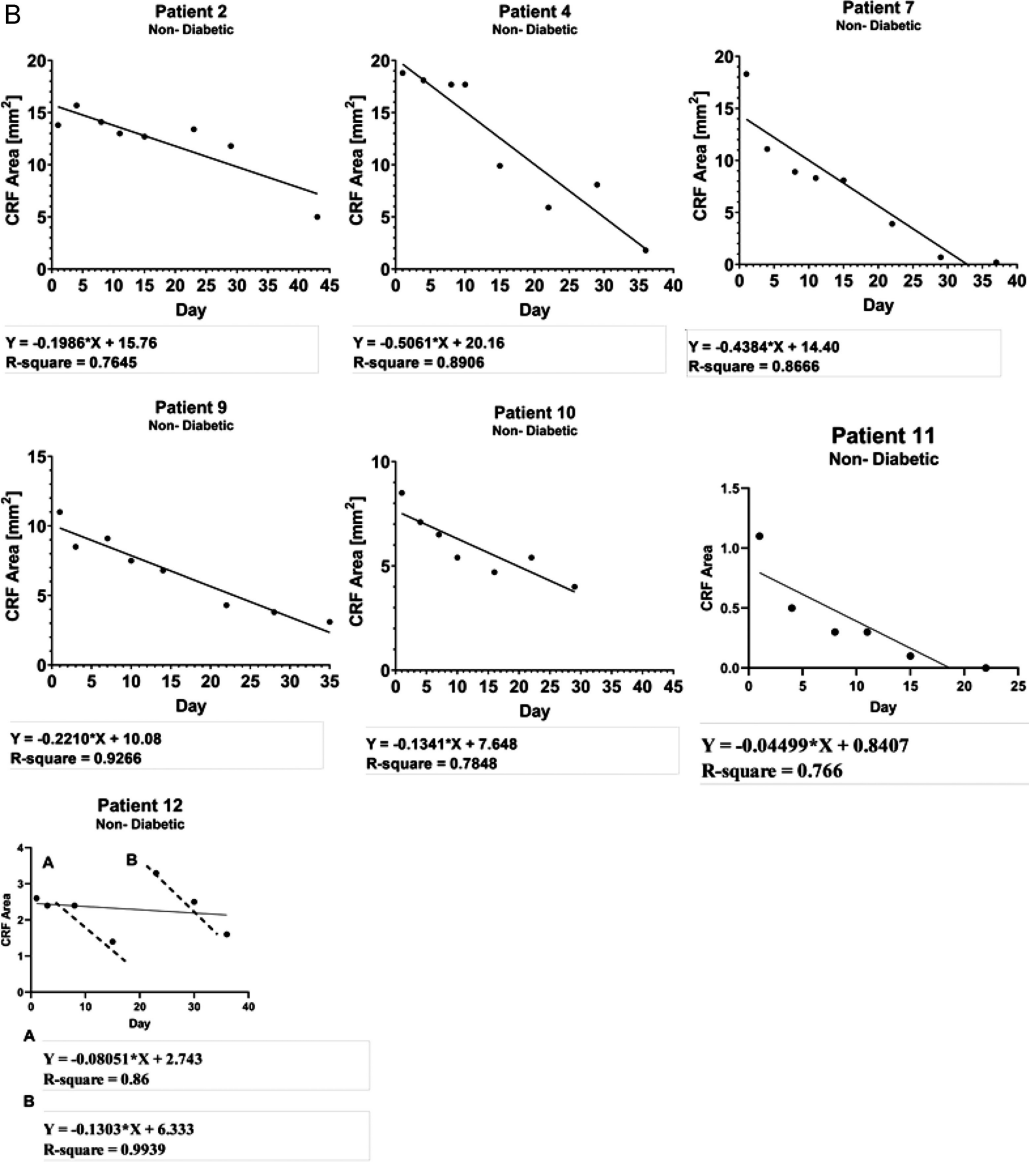
Continued.

### Comparison of Investigator Measurements to Central Reader Measurements

**Figure 4.**
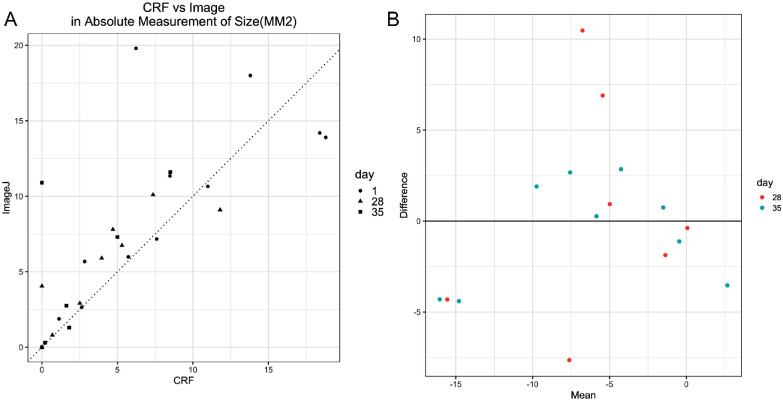
Comparison of wound area measured by CRF and ImageJ analysis. (**A**) Scatter plot of absolute PED area measured by CRF versus ImageJ. The correlation coefficient of the absolute measurements by CRF and Image is 83.22%. (**B**) Bland-Altman plot of mean changes versus difference of two values.

In addition to case report form (CRF) measurements used in the analysis, images of the PEDs were taken and analyzed by a central reader who was masked to the investigator measurements. This analysis showed a statistically significant difference in mean PED area between baseline to day 28 (t(10) = −4.1893, *P* = 0.0009) and between baseline and day 35 (t(8) = −3.9728, *P* = 0.0021). However, there was no significant difference in mean PED area between day 28 and day 35 (t(8) = −0.5685, *P* = 0.2927, not significant [NS]). Scatter plot of the absolute measurement of CRF compared with ImageJ demonstrated a correlation coefficient of 83.22% (Fig. 4A). A Bland-Altman plot was used to evaluate the agreement between the two measurements by plotting the mean of changes detected by two methods versus the difference of the two values (Fig. 4B). There was no pattern between the difference and the mean, however, and the variance between two methods was relatively large.

### Safety

Of the 13 subjects who enrolled in the study, 12 completed treatment and one withdrew early due to reasons unrelated to the study drug. Of the 12 to complete treatment, 10 exhibited no adverse events (AEs). Of the AEs reported, 11 of 16 (68.8%) were mild and included eye pain, tearing, itching, foreign body sensation, burning, photophobia, redness, lightning sensation, flashes, spots in visual field, and swelling. Five of 16 (31.3%) AEs were moderate and included pain, photophobia, tenderness, and ache. No subject experienced a serious adverse event (SAE).

Tonometry data revealed no significant difference between mean baseline and day 28 intraocular pressure (t(10) = −0.054, *P* = 0.958 nor between baseline and day 35 (t(9)) = 0.739, *P* = 0.479.

## Discussion

PEDs can be challenging to manage. Despite a better understanding of the different etiologies of PEDs and the wide range of therapeutic modalities available, some PEDs remain refractory to treatment. Aside from conventional treatments, such as patching, bandage soft contact lenses, preservative-free lubrication, and debridement of the edges of the PED, many experimental treatments have been investigated, including insulin,^41^ substance P,^42^ connexin,^43^ thymosin,^44^ matrix regenerating agents,^45^ and adipose mesenchymal stem cells,^46^ all with varying results.

Biologics, such as autologous serum eye drops and other blood-derived products, including platelets rich in growth factors, have become popular for treating PEDs, as these products contain various cytokines and growth factors, such as EGF, TGF-β1, PDGF, and FGF, that are known to promote corneal epithelial wound healing.^11^^,^^12^ Autologous serum is a preparation taken from the patient in a hospital laboratory that is sometimes diluted and used directly as eye drops in a multidose vial. Use of autologous serum and platelets does not require approval by the US Food and Drug Administration, but its role may be reduced by a lack of insurance coverage.^47^

Recombinant human nerve growth factor (cenergermin 0.002%, Oxervate; Dompe, Milan, Italy) is a newer treatment for neurotrophic keratitis (NK), and has been shown to be effective for decreasing the size of PEDs in stage II NK.^48^^,^^49^ Although this treatment has been found to be effective in several trials, the drug is expensive, and its cost-effectiveness has been questioned.^50^

Amniotic membrane has been used for treating the ocular surface for over 80 years, and it was popularized in the 1990s for its wound healing and anti-inflammatory properties.^51^ Commercially, amniotic membranes are available in dehydrated and cryopreserved forms for placement on the ocular surface, which may be uncomfortable for the patient. ST266 is a unique solution made by proprietary culturing of a selected population of amnion epithelial cells under current good manufacturing practice (cGMP) and collecting their secretions. It contains multiple cytokines and growth factors that act through multiple pathways, such as effects of oxidative stress reduction via SIRT1-mediated mitochondrial function promotion, and pAKT-mediated cell survival signaling.^37^

In the current exploratory phase II multicenter open-label study, PEDs arising from multiple etiologies were enrolled, some previously treated with a varying array of therapies. Eight subjects had previously been treated with amniotic membrane^51^ (PROKERA or PROKERA Slim; BioTissue, Miami, FL, USA) and/or bandage soft contact lens, and four were treated with autologous serum. The etiologies of the PEDs included neurotrophic keratopathy, prior infectious corneal ulcer, and herpes zoster ophthalmicus. Because PEDs may arise from many different causes, ST266, with its complex mixture of wound healing factors that include anti-inflammatory molecules, may address these multiple underlaying etiologies.

Five eyes had complete PED closure during the 28 days of treatment. One of the 5 reopened on day 24 and then closed by day 35 during the post-treatment follow-up period. All eyes demonstrated a decrease in the size of the PED compared with baseline. Of note, in 12 of 12 subjects, the corneal wound was reduced in the area over 28 days. All 12 subjects received topical antibiotics at some time during treatment with ST266. ST266 has been shown in a preclinical rat study to improve healing despite bacterial contamination in the wounds.^36^ This may explain, in part, why all PEDs decreased in size over 4 weeks of treatment. At the 1-week post-treatment evaluation visit (day 35) during which no ST266 was given, healing appeared to be sustained after treatment was discontinued. Importantly, in all but one of 11 eyes at 1-week post-treatment (day 35), the defect was smaller in size than at day 28. One subject had a longstanding PED, which was present for 393 days prior to enrollment in the study. In this eye, ST266 treatment led to a smaller PED size by day 28 (30% smaller compared with baseline) but was 12% larger than baseline 1 week after treatment ended. This is likely related to the underlying condition that initially resulted in the longstanding PED and suggests that a longer treatment period is necessary for complete healing. Indeed, it has been shown that the longer a PED has been open, the longer it takes to heal.^9^

The data from this study suggest that a general reduction in the average PED size is present, but there are considerable fluctuations in measurements for some eyes. The descriptive pattern of PED size across time may indicate a varied response to ST266 treatment. For example, observation of PED area size versus time in patients 2, 4, 7, 9, 10, and 11 showed a strong linear trend suggesting a constant rate of re-epithelization in response to treatment (see Fig. 3B). Notably, these six patients with linear-like re-epithelization rates were non-diabetic, whereas the other five patients were diabetic and showed a nonlinear re-epithelization response to ST266 treatment. Further study with a larger number of eyes will be needed to evaluate this observation.

The technique for measurement of PEDs has been a subject of debate.^52^^,^^53^ Measurement of irregularly shaped lesions by taking the longest measurement multiplied by the longest perpendicular measurement has been well-described, but it does have limitations of accuracy. Correction with methods previously used in chronic pressure sores were used to negate some of this variance.^40^ The use of ImageJ analysis with a masked central reader may allow for more accurate calculations of the area, but it requires that photography protocols be precisely followed. The scatterplot comparing the absolute value of two measurement techniques in the study yielded a correlation coefficient of 83.22%, which suggests acceptable correlation between the two measurement methods. However, the variance of the difference between the two methods appears to be relatively large.

A limitation of this study is the lack of a control group. As such, it is not possible to exclude the fact that some of the improvement seen with ST266 may have happened even without the investigational drug. In addition, the heterogeneous etiologies for the PEDs also limits generalizability. However, this was intended to be an open-label study to try to detect an effect. A phase IIb randomized controlled trial is in the process of being activated in which more eyes will be enrolled, and a placebo arm will be included. In this upcoming study, the condition of the stroma (scarring and melting), as well as the endothelial cell counts (ECC) will also be assessed (no melting occurred in this current study, but scarring and ECC were not measured).

Despite the small size of this trial, the results of this prospective open-label study suggest that ST266 may offer considerable benefit in the treatment of PEDs through reduction in the area of the defect. The promising reduction over just 4 weeks of treatment suggests that a longer treatment period may be needed to reach full closure, especially for patients with longstanding PEDs. To explore this, a randomized, double-masked, and placebo-controlled study is being designed to further assess the efficacy of ST266 in treating PEDs.
